# Doing more with less: The use of non-invasive ventilatory support in a resource-limited setting

**DOI:** 10.1371/journal.pone.0281552

**Published:** 2023-02-16

**Authors:** Heloise Buys, Tamara Kerbelker, Shirani Naidoo, Zakira Mukuddem-Sablay, Zanele Nxumalo, Rudzani Muloiwa

**Affiliations:** 1 Red Cross War Memorial Children’s Hospital, Cape Town, Western Cape Province, South Africa; 2 Ambulatory and Emergency Paediatrics Division, Red Cross War Memorial Children’s Hospital, Cape Town, South Africa; 3 Department of Paediatrics and Child Health, University of Cape Town, Cape Town, South Africa; Sohag University Faculty of Medicine, EGYPT

## Abstract

**Objectives:**

Bubble CPAP (bCPAP), a non-invasive ventilation modality, has emerged as an intervention that is able to reduce pneumonia-related mortality in children in low resourced settings. Our study primarily aimed to describe a cohort of children who were started on CPAP in the Medical Emergency Unit (MEU) of Red Cross War Memorial Children’s Hospital 2016–2018.

**Methods:**

A retrospective review of a randomly selected sample of paper-based folders was conducted. Children started on bCPAP at MEU were eligible for inclusion. Demographic and clinical data, management, and outcomes regarding admission to PICU, need for invasive ventilation and mortality were documented. Descriptive statistical data were generated for all relevant variables. Percentages depicted frequencies of categorical data while medians with interquartile ranges (IQR) were used to summarise continuous data.

**Results:**

Of 500 children started on bCPAP, 266 (53%) were male; their median age was 3.7 (IQR 1.7–11.3) months and 169 (34%) were moderately to severely underweight-for-age. There were 12 (2%) HIV-infected children; 403 (81%) had received appropriate immunisations for their age; and 119 (24%) were exposed to tobacco smoke at home. The five most common primary reasons for admission were acute respiratory illness, acute gastroenteritis, congestive cardiac failure, sepsis and seizures. Most children, 409 (82%), had no underlying medical condition. Most children, 411 (82%), were managed in high care areas of the general medical wards while 126 (25%) went to PICU. The median time on CPAP was 1.7 (IQR 0.9–2.8) days. The median hospitalisation time was 6 (IQR 4–9) days. Overall, 38 (8%) children required invasive ventilatory support. Overall, 12 (2%) children with a median age of 7.5 (IQR 0.7–14.5) months died, six of whom had an underlying medical condition.

**Conclusions:**

Seventy-five percent of children initiated on bCPAP did not require PICU admission. This form of non-invasive ventilatory support should be considered more widely in the context of limited access to paediatric intensive care units in other African settings.

## Introduction

Large numbers of children die annually from pneumonia and diarrhoeal disease despite the significant advances made with improvements in the Expanded Program on Immunisation (EPI) schedules in many low-middle-income countries (LMIC). In 2021, the United Nations Inter-Agency Group reported that the 2030 Sustainable Development Goal (SDG) targets for <5-mortality would not be met in Sub-Saharan Africa and Southern Asia. The highest death rates globally are seen in in three age categories: neonates, infants and children aged 1–4 years who contribute 33%, 20% and 17% (sum 70%) to the 5.1million largely preventable deaths, with pneumonia with acute respiratory failure, diarrhoeal disease and malaria being the top three killers outside of the neonatal age group [1]. There are many children who present with acute undifferentiated respiratory distress from acute respiratory infection with respiratory failure-, or acute severe asthma defined as single organ failure (SOF) with only respiratory dysfunction, or multiorgan failure (MOF)/dysfunction encompassing respiratory failure +/- haemodynamic instability, neurological emergencies [2], congestive heart failure, severe dehydration with metabolic acidosis from diarrhoeal disease, severe kussmaul respiration from metabolic complications including sepsis and poisoning and the group of infants and children who have inadequate respiratory effort and who may present with apnoea, and children with co-morbidities such as malnutrition, HIV exposure or infection, neuro-disability, congenital heart diseases [3]. Red Cross War Memorial Children’s Hospital (RCWMCH) utilises the South African Triage Scale triage tool (SATS) which incorporates the WHO Emergency Triage Assessment and Treatment (ETAT) as its first screen with a triage early warning score (TEWS) developed for use in adults and children. This tool identifies the patients requiring emergency care [1, 4–6].

Non-invasive ventilation (NIV) using bubble continuous positive airway pressure (bCPAP) support has been used for several decades to assist children from varied settings with respiratory diseases. Numerous studies from high income settings have shown its efficacy and benefits in children with acute respiratory failure [7–9]. A panel of experts from the 2017 Paediatric Mechanical Ventilation Consensus Conference (PEMVECC) recommended that NIV should be considered before turning to mechanical ventilation but without delaying MV and noted that it is increasingly recognised as beneficial in acute respiratory failure in patients with a variety of illnesses e.g., neuromuscular disease, severe asthma, post-op cardiac surgery, weaning from mechanical ventilation [10]. Furthermore, both CPAP and high flow nasal cannula (HFNC) therapy have been found to be useful as post-extubation non-invasive respiratory support for children with heterogenous clinical conditions requiring respiratory support- both single and multi-organ failure [11]. The use of NIV bCPAP support in neonates is better established in many countries and at various tiers of the health care service including resource-limited settings [2, 12–14]. Bubble CPAP is therefore an important and cost-effective option in resource-limited settings to provide non-invasive respiratory support for better outcomes for neonates with respiratory distress as well as for children with single organ dysfunction with severe hypoxic pneumonia, who when supported by bCPAP, have better outcomes (survival) compared to those given low flow oxygen therapy. [15]. The use of NIV in paediatrics in high income settings also is routinely undertaken in paediatric intensive care (PICU) settings. PICU space is always at a premium in most low resourced health systems. It is this pressure that led to the realisation that something different needed to be done in resource-limited settings to effectively manage children with acute cardiorespiratory distress in high care units outside of PICU where the staff-to-patient ratios fall well below those within intensive care units. CPAP is an important NIV-mode capable of unloading the burden of excessive respiratory muscle work of breathing (WOB), improvement of respiratory distress and outcomes [16]. Bubble CPAP has been described as a safe and efficient form of CPAP, however whether CPAP is used for children with single organ dysfunction such as severe pneumonia [15, 17] or for neonates with respiratory distress syndrome [12, 18], considerable thought and adequate preparation in terms of leadership, training, equipment, consumables and technical support are key issues so that the NIV support is delivered safely within acceptable standards of care [18–20]. Hence the set-up of bCPAP systems, especially of ‘low-cost devices’ needs to follow recognized standard set-ups which include the short nasal prongs, no dead space and expiratory tube diameter of at least 8mm (i.e., wide-bore), as modifications which deviate from these standard settings result in increased WOB and inadvertent higher levels of CPAP and may be dangerous [16, 18, 19, 21].

This study aimed to describe the characteristics and outcomes of children initiated on bCPAP at the medical emergency unit in those children presenting with acute cardiorespiratory distress requiring respiratory support.

## Methods

### Study design and timelines

A retrospective descriptive cross-sectional study was conducted that involved a case note review of children who were initiated on bCPAP the MEU, from 2016 to 2018.

### Study population and Setting

RCWMCH provides high care and paediatric intensive care support to a population of about 1.5 million children younger than 14-years-of age outside the newborn period. Specialised newborn care is provided elsewhere. Although the population of children served by RCWMCH has exponentially increased over the last two decades, the number of PICU beds have remained static. The pathway of children accessing care at the study site has previously been described as quite complex, most children coming in from impoverished communities- access to emergency care is provided at local nurse-led clinics, community health clinics, and district hospitals, though a fair proportion come directly from home in hired transport or some are able to engage the overstretched emergency medical ambulance services [22].

#### Data collection

All children who were started on bCPAP in the MEU from 1^st^January 2016-December 2018 were eligible for inclusion. Their folder numbers were abstracted from the unit’s paper-based register and scanned into an excel sheet list, two of the study staff were responsible for retrieving the folders from the medical records department and entering the data into a paper-based case report form; the PI was responsible for overseeing the data entry into a bespoke de-identified electronic excel datasheet and for checking the entries.

Excluded were children started on bCPAP in other areas within the hospital including PICU, children on home ventilation such as bi-level positive airway pressure (BiPAP) as well as children with tracheostomies and those intubated in the emergency unit as the first-line mode of respiratory support.

### Procedures

The study was preceded by the introduction, in 2012, of iterative cycles of training for nurses and doctors regarding the use of nasal prong bCPAP, and the procurement of standardised equipment for use at our facility (Fisher and Paykel® bCPAP with appropriate circuitry). The decision to commence nasal prong bCPAP was made by the RCWMCH emergency medical paediatric team. Children eligible for inclusion were those started on and stabilised on Fisher and Paykel® bubble bCPAP via nasal trunk prongs applied with hydrocolloid protective film around the nostrils and philtrum to minimise nasal trauma (with scheduled nursing checks) and a flow rate of about 8L/ minute (range 6-12L/minute, dependant on adequate bubbling being evident), starting pressures of 5cm water and FiO2 of 100%; thereafter titrated fractional inspired oxygen (FiO2) concentrations to maintain saturations according to WHO recommendations- the targeted SpO2 for children with MOF should be above 94% in order to meet the tissue oxygen consumption demands in children with severe pathophysiological conditions. In our setting, children not improving on 7-8cm pressures bCPAP, and/or whose FiO2 needs were ≥60%, or the work of breathing thought to be persistently high, were moved into the PICU for further care.

Scheduled in-service training sessions were conducted after 2012 to maintain skill levels in the use of bCPAP separately for the nurse and doctors. The nurses were trained to check the circuits for leaks, heated humidification settings, water levels in the chambers, condensation rain-out in the circuitry, water pressure levels and how to respond to alarms. Additionally, they were expected to titrate the oxygen concentration targeting SpO2 levels of 94–98% [6, 23]. Fittings were to be safe but secure without undue pressure, correcting displacements in active children and to perform gentle clearance of the nasopharyngeal passages of excess mucus accumulation. The doctors on duty were trained to recognise and monitor for treatment failure at several time points in the day and night and monitor the need for escalation of respiratory support on the one hand and how to go about active weaning on the other hand. Available resources at the study site are described in **S1 File**.

#### Case definitions

Single organ failure (SOF) was defined as only respiratory dysfunction; multiorgan failure (MOF)/dysfunction encompassed respiratory failure + haemodynamic instability (shock, dehydration), neurological emergencies, acute kidney injury [2]. Co-morbidities included conditions such as malnutrition, HIV exposure or infection, neuro-disability, congenital heart diseases.

#### Data analysis

All children meeting inclusion and with none of the exclusion criteria were eligible for analysis. Data from RCWMCH indicated that 30 to 50 children per month received bCPAP over the three-year period under review. We hypothesised that the failure rate of bCPAP in which children would need invasive ventilation for respiratory support would be 10%. A sample of 500 patients would provide a precision that would fall within 3% of the hypothesised 10% bCPAP failure (95% CI 7.37% to 12.63%) which we felt to be acceptable. A random number generator was used to select the included folders.

Descriptive statistical data variables included simple demographics and basic pathological groupings such as age, sex, gestation, birthweight, breastfeeding history, nutritional status, immunisation history, exposure to tobacco smoke at home, primary diagnosis at presentation, any underlying long-term health condition, disposition, outcomes such as need for PICU admission, escalation to invasive ventilation, duration of CPAP support, duration of hospitalisation, complications related to CPAP and any mortality. HIV data was captured as well to help provide complete epidemiological profiling of the study population. Categorical data were presented as frequencies and percentages. Since all continuous data were skewed medians with interquartile ranges (IQR) were used to describe the distribution of the data. Failure of bCPAP was examined across clinical diagnostic groups, indications for bCPAP as well as demographic characteristics. All analyses were done using Stata Statistical Software Release 16 (StataCorp LP, College Station, TX).

### Ethical and legal considerations

The study was approved by the Human Research Ethics Committee of the University of Cape Town and Red Cross War Memorial Children’s Hospital Administration (HREC 764/2018). As this was a retrospective study spanning three years, a waiver of individual consent was also approved. All information pertaining to patients’ credentials was de-identified and protected by the principal investigator to guarantee anonymity.

## Results

**Fig 1:**
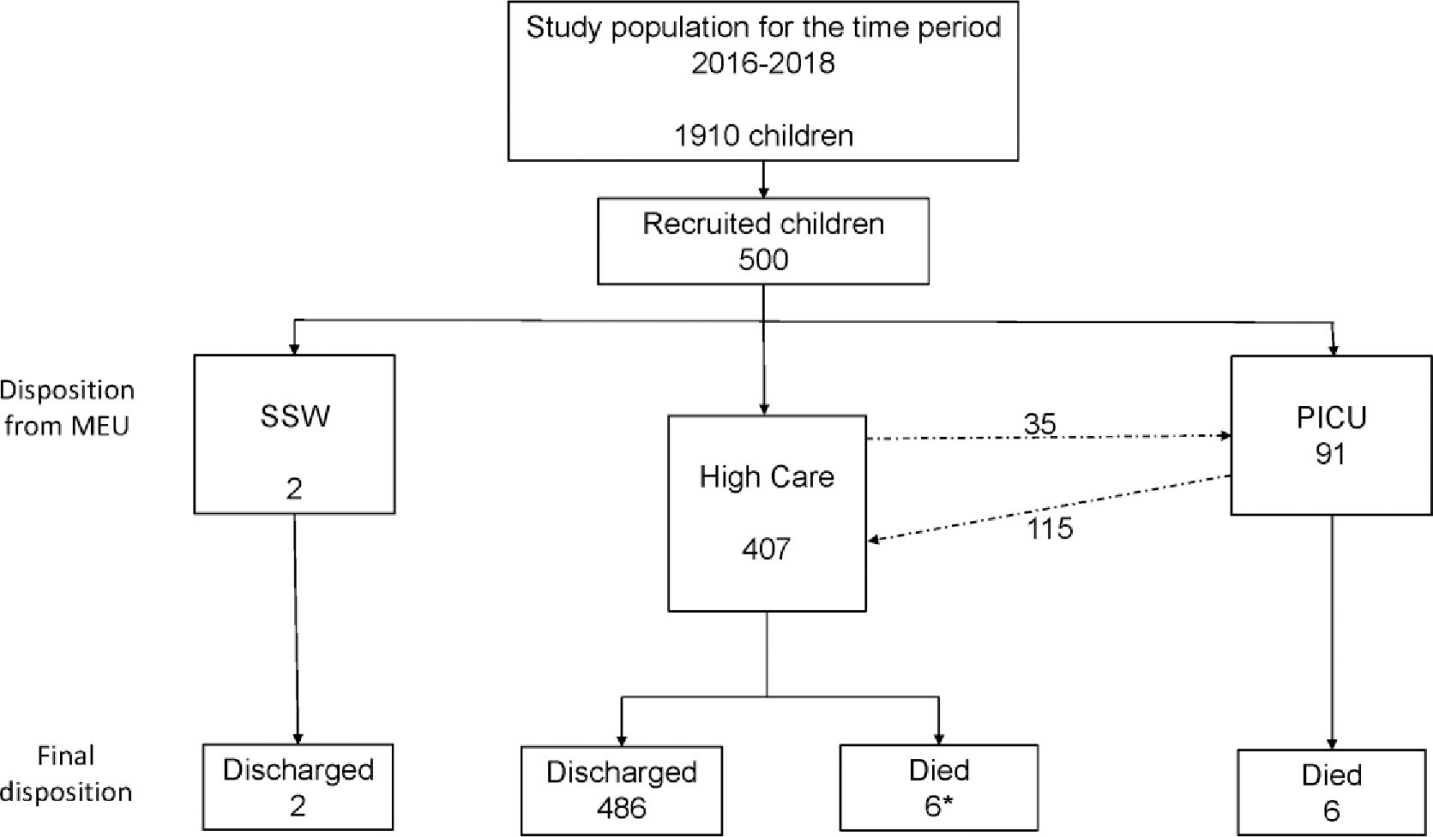
The study recruitment and outcome by initial disposal. * 5 included in the 35 transferred to PICU and died in PICU; MEU- medical emergency unit; SSW- short stay ward; PICU- paediatric intensive care unit.

Of the 1 910 children started on bCPAP during the study period 500 were selected for analysis. The recruitment and flow of patients throughout the study period is depicted in **Fig 1.**

Of the 500 children started on CPAP included in the analysis, 266 (53%) were male. The median age of the study group was 3.7 (IQR 1.7–11.3) months, while the median WAZ-score was -1.2 (IQR, -2.5 to 0.1). The cohort had a median birth weight of 2.89 (IQR 2.32–3.29) kg and 169 (34%) were moderately-severely underweight-for-age children. Most of the patients had been appropriately immunised for their age {401 (80%)}; and 119 (24%) were exposed to tobacco smoke at home. There were 12 (2%) HIV-infected children, and their median age was 2.8 (IQR 1.6–5.5) months, eight were male. Four of the children were on antiretroviral therapy (ART) prior to admission, however, there were no recent HIV viral load or CD4 counts available for any of the four; the rest of the infected children were diagnosed during this index admission via the ED, none had viral load or CD4 count measurements during the acute admission episode. Half of them were being breastfed on admission, six were moderately to severely underweight-for-age, there were no other nutritional indicators available. Ten presented with single organ failure- severe pneumonia, one of whom also had disseminated TB; one with diarrhoeal disease and severe dehydration, and one with circulatory shock and MOF—this child was a neonate who was not on ART and died in the PICU. Disseminated toxoplasmosis was found at autopsy. Two of the 12 were admitted to the PICU, though none received MV. The median time on CPAP was 1.75 (IQR 1.2 to 2.85) days, their mean length of hospital stay was 10.5 days ± 6 days.

The five most common primary reasons for admission were acute respiratory illness, acute gastroenteritis, congestive cardiac failure, sepsis, and seizures **Table 1**.

**Table 1:** Baseline characteristics of children started bCPAP for respiratory support (N = 500).

Variable	n/N	(%)
Sex		
Male	266	(53)
Age category		
neonate	57	(11)
≤3 months	226	(45)
≤12 months	376	(77)
Birth weight kg		
Low birth weight (<2.5kg)	151	(32)
Preterm (<37 weeks)	141	(28)
Immunisation		
IUTD	403	(81)
Missing ≥1	83	(16)
Unknown	16	(3)
Nutritional status		
NWFA	331	(66)
moderate-to-severe UWFA	169	(34)
HIV status		
Infected	12	(2)
HIV-exposed uninfected	124	(25)
HIV-uninfected, unexposed	360	(72)
Unknown	4	(1)
Home tobacco smoke exposure		
yes	119	(24)
no	76	(15)
unknown	305	(61)

NIV- non-invasive ventilation; bCPAP- bubble continuous positive pressure ventilation; IUTD- immunisations up to date for age; WAZ score- weight-for-age-z-score; NWFA- normal weight-for-age; UWFA- underweight-for-age

The SATS triage tool identified all children as requiring immediate medical treatment and/or resuscitation. Eighteen percent (n = 91) of children had a chronic underlying medical condition with the two most common being congenital cardiac abnormality and complex congenital syndrome. The most common presenting complaint was severe respiratory distress. The top three primary conditions were acute respiratory infection, acute gastroenteritis and sepsis **Table 2**.

**Table 2:** Clinical characteristics of the children started on bCPAP outside of PICU (N = 500).

Variable	N	(%)
Referred	360	(72)
Triage code^$^		
Red	495	(99)
Orange	5	(1)
Chronic comorbidity		
Nil	409	(82)
Cardiac	30	(6)
Congenital syndrome	19	(4)
Cerebral palsy	5	(1)
Chronic asthma	4	(1)
Chronic lung disease	3	(1)
Other miscellaneous	46	(9)
Presenting complaint at triage		
Severe respiratory distress/apnoea	451	(90)
Diarrhoea with severe dehydration	22	(4)
Coma and Convulsions	14	(3)
Circulatory shock	11	(2)
Other Red	6	(1)
Primary diagnosis		
SOF		
Acute respiratory illness	433	(87)
MOF:		
Acute gastroenteritis	25	(5)
Sepsis	19	(4)
Congestive cardiac failure	12	(2)
Seizures	7	(1)
Other^#^	4	(1)

bCPAP- bubble continuous positive airway pressure; PICU- paediatric intensive care unit

$—Paediatric South African Triage Scale 2012 (with ETAT); Other

#—encephalopathy, poisoning, non-fatal drowning, acute surgical abdomen; SOF-single organ failure; MOF- multiorgan failure (respiratory dysfunction + haemodynamic instability (shock, dehydration, anaemia), reduced level of consciousness)

Of all the 126 children who were admitted to the PICU at any time, 91 (72%) children were admitted directly to PICU from the MEU, and a further 35 children were transferred to PICU from the high care units. Intubation and invasive ventilatory support were required in 38 (8%) of all patients. The median time spent on bCPAP was 1.7 (IQR 0.9–2.8) days and the median length of hospitalisation was 6 (IQR 4–9) days **Table 3**.

**Table 3:** Outcomes of the study population commenced on bCPAP in the MEU (N = 500).

	n	(%)
Ward disposition from ED		
General ward high dependency area	407	(81)
PICU	91	(18)
SSW	2	(1)
Number admitted to PICU anytime	126	(25)
Went to PICU from MEU	91	(18)
Went to PICU from ward	35	(7)
Required IPPV in MEU	10	(2)
Required IPPV ever	38	(8)
Complications		
Pneumothorax	2	(0.004)
Died during current admission	12	(2)

MEU- Medical Emergency Unit; bCPAP- bCPAP- bubble continuous positive airway pressure; PICU- paediatric intensive care unit; SSW- short stay ward; IPPV- invasive positive pressure ventilation

There were 32 (25%) children out of the 126 admitted to PICU who had a chronic medical condition compared to 60 (16.4%) of the 374 who did not require admission to PICU; p = 0.02. Similarly, 11 (8.7%) of those requiring PICU died compared to one (0.3%) in those not admitted to the PICU; p<0.0001 **Table 4**.

**Table 4:** Comparison of children by disposition ward and type of respiratory support.

	Disposition ward			Respiratory support		
Variable	Ward	PICU	p-value	No IPPV*	IPPV	p-value
	n = 374	n = 126		n = 462	n = 38	
	n (%)	n (%)		n (%)	n (%)	
Sex (male)	201 (53.7)	65 (51.6)	0.68	250 (54)	16 (42)	0.15
Chronic underlying condition	60 (16)	32 (25.4)	0.02	83 (18)	9 (23.7)	0.38
Alive at discharge	373 (99.7)	115 (91.3)	<0.0001	456(98.7)	32(84.2)	<0.0001

PICU- paediatric intensive care unit

*all children on bCPAP- bubble continuous positive airways pressure; IPPV- invasive ventilation

**Fig 2:**
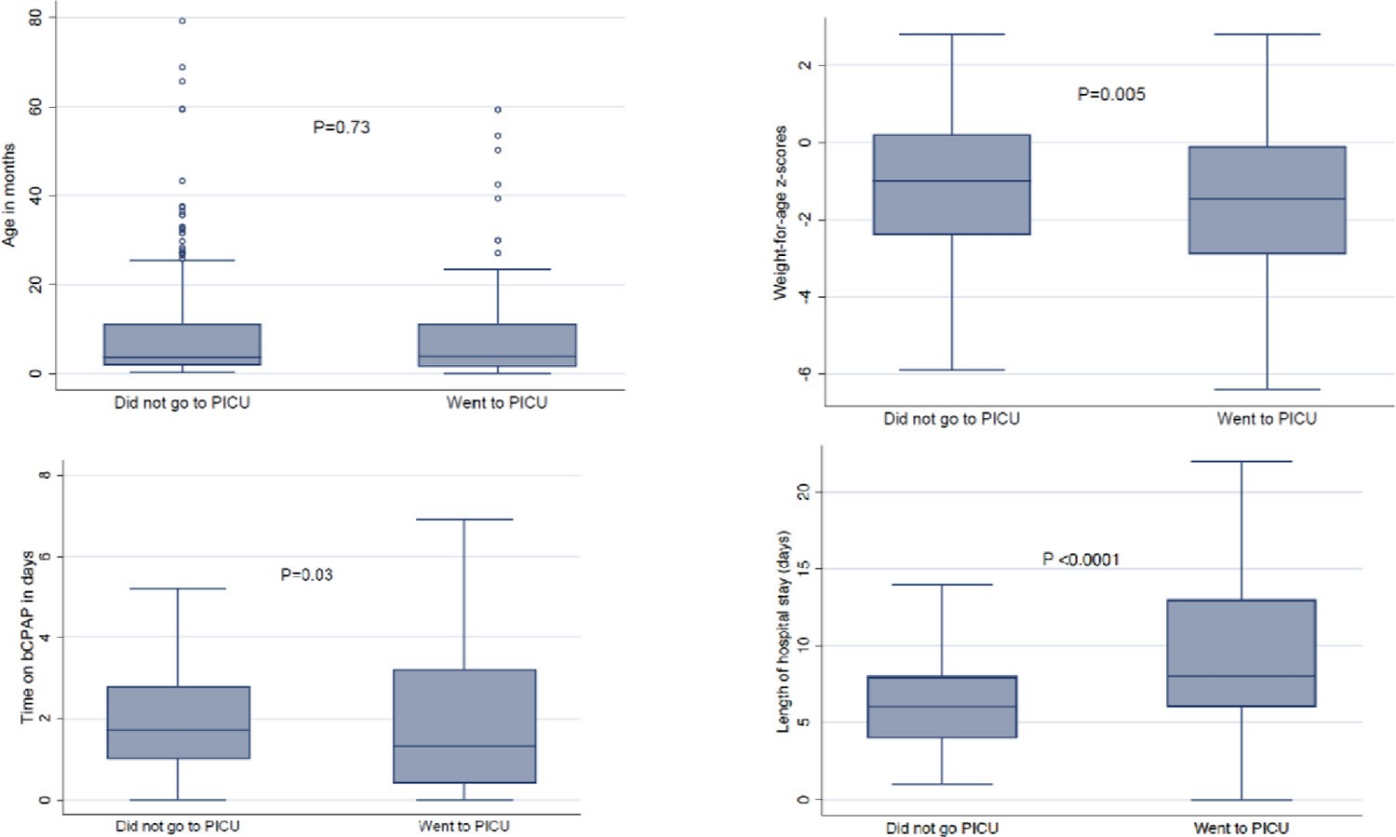
Comparison of children who were admitted to PICU vs. those who were not. PICU-paediatric intensive care unit; bCPAP- bubble continuous positive airway pressure.

Children admitted to the PICU were on bCPAP for a median of 1.3 (IQR 0.4–2.9) days compared to those not requiring PICU with median of 1.7 (IQR 1–2.8) days; p = 0.03. This most likely reflects the need for intubation and invasive ventilation. The same children who required ICU were hospitalised for a median of 8 (IQR 4–8) days compared to those not requiring PICU who were admitted for a median of 6 (IQR 4–8) days; p<0.0001 **Fig 2**.

**Fig 3:**
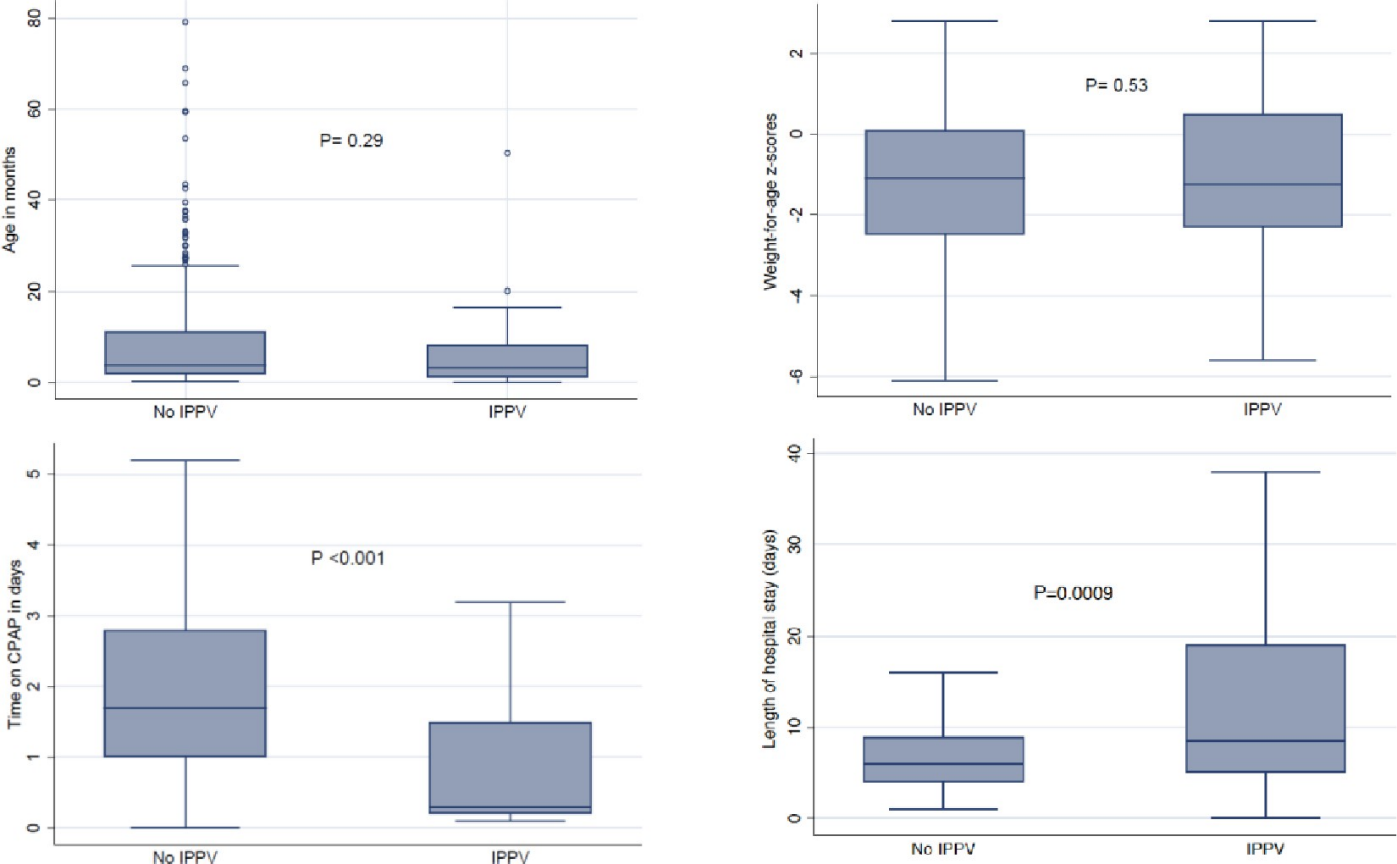
Comparison of children requiring intubation vs. those who did not. IPPV- invasive ventilation; bCPAP- bubble continuous positive airways pressure.

Children requiring intubation and IPPV had the highest mortality and had longer length of hospitalisation, compared to those who did not require intubation, p<0.0001 and p = 0.0009 respectively **Table 4 and Fig 3**.

### Mortality

A total of 12 (2%) children in the study group died; 11 of the 12 children died in the PICU. Six of the children who died had a serious coexisting underlying health condition while another three had overwhelming sepsis from pneumococcus, meningococcus, and *Escherichia coli* respectively. Acute respiratory infection and overwhelming sepsis was the final cause of death in nine (75%) of the children **Table 5**.

**Table 5:** Characteristics of the 12 children who died.

Variable		
n (%)	12/500	(2)
Male sex	5	(42)
Median age in months (IQR)	7.5	(0.6–14.5)
IUTD	10	(83)
WAZ-score (IQR)	-0.1	(-1.9 to 0.5)
HIV-infected	1	(8)
Home tobacco smoke exposed	2	(17)
Time on bCPAP in days (IQR)	0.5	(0.3–3.5)
LOS in days (IQR)	3.5	(1–16.5)
Chronic comorbidity y/n	6	(50)
Cause of death		
Pneumonia	5	(43)
Sepsis	3	(25)
Meningitis	1	(8)
Myocarditis	1	(8)
CCF, Complex congenital heart	1	(8)
Inborn error of metabolism	1	(8)

QR-interquartile range; IUTD-received immunisations appropriate for age; WAZ-score- weight for age z-score; bCPAP- bubble continuous positive airway pressure support; LOS- length of stay; y/n- yes/no; CCF- congestive cardiac failure

## Discussion

This study provides a review of the use of non-invasive respiratory support in a large cohort of children in an African tertiary paediatric hospital. Five hundred children identified as needing emergency care were commenced on bCPAP in the medical emergency unit for undifferentiated respiratory distress with favourable results. The mortality rate in our cohort was low (2%) and occurred associated with overwhelming sepsis and multi-organ-dysfunction. In this study, 75% of children initiated on bCPAP did not require mandatory admission to a PICU, this is in comparison to our previous policy of all children being admitted to the PICU for bCPAP support. In order to provide bCPAP in this safe and effective manner, the HCUs do require adequate numbers of suitably trained nursing staff. The cost implications of this are enormous in these times of austerity, and of huge significance to LMICs.

It is thought that NIV works by improving alveolar gas exchange by providing support to the compliant and immature paediatric chest wall, reducing the work of breathing and increasing functional residual capacity [24]. This provides relief in acute respiratory distress and allows time for natural resolution with or without adjunctive care.

There are scarce randomised controlled trials (RCT) on bCPAP. One such study from Bangladesh showed that bubble bCPAP reduced pneumonia-related mortality in children <5years of age compared to traditional low-flow oxygen therapy, RR 0·25, 95% CI 0·07–0·89; p = 0·022 [15]. A multicentre RCT from Australia and New Zealand involving 1 472 infants with hypoxaemic bronchiolitis found that high-flow oxygen therapy was more beneficial than standard nasal prong oxygen therapy care, NNT = 9 [25]. The benefits of bCPAP have been realised in other developing countries in a variety of conditions including viral pneumonia, bronchiolitis, asthma, dengue fever and other undifferentiated respiratory distress syndromes [13, 17, 26–30].

Neonatal units in LMICs including South Africa have been using NIV to stabilise newborns with apnoea of prematurity and neonatal respiratory distress syndrome for longer than paediatric units. Recently, a rural district South African hospital reported that NIV can also be applied safely in rural settings for mild to moderate acute neonatal respiratory distress syndrome, and in so doing saw a 14% reduction in transfer rates to secondary or tertiary hospitals [14]. In recognising the potential for CPAP to reduce mortality in LMICs, resources such as staffing and their training, recognised equipment and their consumables, clinical engineering, technical support and stable power availability day and night are important factors to be considered when planning the introduction of CPAP and NIV [12].

In keeping with these outcome studies, our study indicated that bCPAP offers good and safe support particularly to infants with SOF- those with viral bronchiolitis/pneumonia. A randomised controlled trial by Yanez et al., involving 50 children admitted to paediatric intensive care units with acute respiratory failure in Santiago, Argentina, showed improvements in respiratory rates and effort and heart rates by the second hour of treatment following admission (*p* = 0.004 and *p* = 0.0009, respectively) [31]. The context and findings are similar to our study. Our facility has a wide drainage area, and optimising referrals is part of obtaining good outcomes. The evolution of transport ventilators to include a mode of safe delivery of bCPAP has extended the benefits of NIV support to children during referral up to tertiary units, removing the dangers of failed intubation at community level. Our study did not report on this, but our anecdotal experience strongly suggests that NIV has added to the safety of the ambulance transfer of critically ill children to tertiary units. The paediatric literature is not clear on how to identify the patient groups with undifferentiated respiratory distress who are unsuitable for bCPAP or likely to fail NIV. The Paediatric Mechanical Ventilation Consensus Conference of 2017 recommendations state that CPAP be considered prior to turning to MV for support in mixed cardiorespiratory failure, though not to delay intubation [10]. Treatment of children with respiratory dysfunction managed with bCPAP, presenting with further organ dysfunctions (MOF) may have a higher mortality risk than children with SOF [2, 32]. For adult patients, Mehta and Hill identified that normal level of consciousness, moderate respiratory acidosis rather than severe distress, younger age (adults) and observed improvement within two hours were predictors of success [33]. Data from paediatric studies are sparse, however one study involving 114 children from neonates to 16 years of age, showed that ARDS (acute respiratory distress syndrome) and a high PELOD (pediatric logistic organ dysfunction) score were predictors of failure in children admitted to a PICU in France [34]. A further two small observational studies conducted in Malawi [2]and Papua New Guinea [32] identified the following risk factors for bCPAP failure: multi-organ failure, and co-morbidities such as HIV exposure and infection, and nutritional status in many very low- resource contexts is a further risk factor for mortality among critically ill children. The essence of our study is premised on a move away from managing all children on NIV in PICU, and hence the PELOD score is not applicable to our setting. Six of the 12 children in our study who died had MOF-serious underlying conditions and overwhelming pneumonia and bacterial sepsis. The 38 (8%) children who failed bCPAP and subsequently required intubation and invasive positive pressure ventilation (IPPV), showed clinically detectable increase in cardio-respiratory distress and work of breathing.

Our study also showed that children admitted to PICU or those who required IPPV, had longer hospitalisation and had higher mortality, suggesting that these were the sickest children and decision to intubate and ventilate early may have been something to consider. The factors influencing their outcomes are however likely to be multifactorial, and our study is unable to indicate risk factors for those likely to fail NIV. This could be addressed in future studies.

There are some severe adverse events associated with bCPAP that have been described in case reports, indicating that whilst it is a lower-cost intervention, good clinical care is still required when it is employed -soft tissue injuries, air leaks-pneumothorax and pneumocranium and failed NIV have been described [35]. Our study was not able to adequately describe the full side-effect profile encountered during the use of NIV bCPAP mainly due to poor documentation in the clinical notes. Notably though, there were two children who experienced air leak in the form of pneumothoraces- this type of complication is easy to pick up and requires good clinical vigilance so that the problem can be addressed and resolved timeously.

The study is limited by the retrospective nature of its design which depends on records of clinical data. This has the potential to introduce bias into the study as some data or detail may be unavailable. This is a single centre study but probably the largest and only South African dedicated tertiary children’s hospital making extensive use of non-invasive respiratory support delivered outside of PICU for children requiring tertiary hospital care. A further limitation is the fact that the basis for clinical judgment in making important management changes is not always well captured in the clinical notes. Milési [36] and Duke [32] have also outlined technical considerations, physiological understanding and possible solutions to users of CPAP and HFNC. Several low-cost bubble CPAP devices are available. Although this is a low-cost life-saving intervention there are some commitments to be made even in LMICs to make this a sustainable investment. From a quality assurance point of view, it is crucial that these devices follow internationally acceptable standards [18, 19, 36] including the procurement and maintenance of equipment including the correct respiratory circuits, ensuring adequate flow in relation to the patient’s size/weight. [36] the ability to adapt FIO2 whilst providing adequately humidified air- oxygen flow and finally, ensuring reliable electricity back-up and maintenance arrangements in order to guarantee the safe function of these devices. Despite these associated costs, on a very positive note—the returns on investment are huge for child health and survival compared to the alternative of using the PICU to provide support for all children with severe respiratory distress.

This is a large sample size from a single centre, and the data collected potentially provided information on which children benefit most from bCPAP included: the number commenced on bCPAP, primary diagnosis on admission, time on bCPAP (days), length-of-stay (LOS), the number of children, started on bCPAP, who did not improve or deteriorated and needed IPPV (i.e. failed bCPAP support), those who died and the cause of death where known and any documented complications thought to be directly related to the bCPAP support used e.g. air leaks, soft tissue injury.

## Conclusions

This study showed that in a large paediatric University Hospital in South Africa bCPAP could be safely initiated in the emergency department. For children with improving respiratory function bCPAP could be safely continued outside a well-equipped PICU in a clinical area staffed by adequate numbers of health workers trained in the use of NIV and essential paediatric emergency and critical care. This form of non-invasive ventilatory support should be considered more widely in the context of limited access to paediatric intensive care units in other resource-constrained settings.

## Supporting information

S1 File The level of care provided in the ED vs ward high care area vs PICU at the study site. (DOCX)

S2 File (PDF)
