# Supplementary material for: Doing more with less: The use of non-invasive ventilatory support in a resource-limited setting
Source: PLoS One. 2023 Feb 16;18(2):e0281552. doi: 10.1371/journal.pone.0281552 (PMC9934338; doi:10.1371/journal.pone.0281552)
Supplement: S1 File — (DOCX) [file pone.0281552.s001.docx]

**Supplement 1:The level of care provided in the ED vs Ward High Care Area vs PICU at the study site**

| **Capacity** | **ED** | **Ward-level high care** | **PICU** |
| --- | --- | --- | --- |
| A_B_C_D resuscitation | Yes | Yes | Yes |
| Multiparameter monitoring (including SpO2) | Yes | Yes | Yes |
| Piped wall oxygen supply with blender control | Yes | Yes | Yes |
| NG tube insertion and feeding.  Breast feeding also encouraged | Yes, though feeding deferred to ward-level care | Yes | Yes |
| Standardised bCPAP, heated humidification, disposable circuitry and consumables | Yes | Yes | Yes |
| HFNC | Yes | Yes | Yes |
| MV | Yes- short term only (pre-PICU) | No (intubation and transfer to PICU) | Yes |
| HFO | No | No | Yes |
| Fluid resuscitation including BTF | Yes | Yes | Yes |
| Blood bank on site | Yes | | |
| Infusion pumps and syringes | Yes | Yes | Yes |
| Inotrope infusion | Yes- peripheral | Yes- peripheral | Yes- central |
| Invasive monitoring | no | no | Yes |
| POC blood gas, acid-base, haemoglobin, glucose and electrolyte analyser | Yes | No- access provided via ED or PICU | Yes |
| POCUS | Yes | No | Yes |
| Mobile X-ray | Yes | Yes- infrequent | Yes |
| Emergency access to CT scanner | Yes | Yes | Yes |
| Immediate antibiotic initiation and management of severe infections including isolation | Yes  limited isolation | Yes | Yes |
| Bedside parent | Yes- one | Yes | Visits only |
| Paediatrician | Yes- attending onsite 08-17:00, then on call | Yes-twice daily round, then on call | Intensivist attending, then on call |
| On-site staff |  |  |  |
| 08-17:00 | Paediatric registrar | Paediatric registrar | Paediatric registrar |
| 17-24:00 | Paediatric senior registrar | Paediatric registrar on multi-ward (including EC after 24:00) cover  plus medical officer | Dedicated paediatric registrar x 2 |
| 24:00-08:00 | Medical officer x 2 |  |  |
| Nurse: patient ratio | 2-3:8 | 1:5 | 1:1 |
| Clinical technician support for all wards | Yes | Yes | Yes |
| Emergency power generator | Yes | Yes | Yes |

Legend: A_B_C_D- Airway_Breathing_Circulation_Disability; ED- emergency department; PICU- paediatric intensive care unit; NG- nasogastric; bCPAP- bubble continuous positive airway pressure; HFNC- high flow nasal canula; MV- mechanical ventilation; HFO- high frequency oscillation; BTF- blood transfusion; POC- point-of-care; POCUS- point of care ultrasound; CT- computed tomography
