# Supplementary material for: Doing more with less: The use of non-invasive ventilatory support in a resource-limited setting
Source: PLoS One. 2023 Feb 16;18(2):e0281552. doi: 10.1371/journal.pone.0281552 (PMC9934338; doi:10.1371/journal.pone.0281552)
Supplement: S2 File — (PDF) [file pone.0281552.s002.pdf]

```
 ___  ____  ____  ____  ____(R)
/__    /   ____/   /   ____/
___/   /   /___/   /   /___/
  Statistics/Data analysis
```

User: Heloise Buys
Project: CPAP Study: comparison of children by disposition and resp support

```
  ___  ____  ____  ____  ____ (R)
 /__    /   ____/   /   ____/
___/   /   /___/   /   /___/   16.1
  Statistics/Data analysis
```

Copyright 1985-2019 StataCorp LLC
StataCorp
4905 Lakeway Drive
College Station, Texas 77845 USA
800-STATA-PC   https://www.stata.com
979-696-4600   stata@stata.com
979-696-4601 (fax)

Stata license: Single-user perpetual
Serial number: 301606206653
Licensed to: Heloise Buys
University of Cape Town

Notes:
1. Unicode is supported; see help unicode_advice.

1 . *(55 variables, 500 observations pasted into data editor)

2 . *CPAP Study

3 .
4 . tab sex

sex	Freq.	Percent	Cum.
0	**234**	**46.80**	**46.80**
1	**266**	**53.20**	**100.00**
Total	**500**	**100.00**	

5 .
6 . *Exploring the age groups of the affected children

7 .
8 . swilk agem

Shapiro-Wilk W test for normal data

Variable	Obs	W	V	z	Prob>z
agem	**500**	**0.67427**	**109.573**	**11.290**	**0.00000**

9 .
10 . tabstat agem, stats (n mean med sd p25 p75 min max)

variable	N	mean	p50	sd	p25	p75	min	max
agem	**500**	**8.20888**	**3.72**	**10.9322**	**1.74**	**11.25**	**.07**	**79.18**

11 .
12 . *Nutritional status and weight-for-age Z-score (waz)

13 .
14 . *egen waz=zanthro(mass,wa,US), xvar(agem) gender(sex) gencode(m=1, f=0) ageunit(month) nocutoff

15 .

```
16 . * Done and copied and added to excel database as waz

17 .
18 . swilk waz
```

Shapiro-Wilk W test for normal data

Variable	Obs	W	V	z	Prob>z
waz	**500**	**0.96411**	**12.074**	**5.988**	**0.00000**

```
19 .
20 . tabstat waz, stats (n mean med sd p25 p75 min max )
```

variable	N	mean	p50	sd	p25	p75	min	max
waz	**500**	**-1.3396**	**-1.2**	**1.982689**	**-2.5**	**.1**	**-11.6**	**2.8**

```
21 .
22 . recode waz -11.7/-3=3 -2.999999999/-2=2 -1.99999999999/10=1
   (waz: 495 changes made)

23 .
24 . label define wazlbl  1 "Normal" 2 "Moderate Underweight" 3 "Severe Underweight"

25 .
26 . label values waz wazlbl

27 .
28 . tab waz
```

waz	Freq.	Percent	Cum.
Normal	**331**	**66.20**	**66.20**
Moderate Underweight	**75**	**15.00**	**81.20**
Severe Underweight	**94**	**18.80**	**100.00**
Total	**500**	**100.00**	

```
29 .
30 . *disposition ward where 4=picu, 1,2,and 5 =other highcare wards

31 .
32 . tab ward
```

ward	Freq.	Percent	Cum.
1	**2**	**0.40**	**0.40**
2	**360**	**72.00**	**72.40**
4	**91**	**18.20**	**90.60**
5	**47**	**9.40**	**100.00**
Total	**500**	**100.00**	

```
33 .
34 . *but wenticu includes all who went to icu

35 .
36 . tab wenticu
```

wenticu	Freq.	Percent	Cum.
0	**374**	**74.80**	**74.80**
1	**126**	**25.20**	**100.00**
Total	**500**	**100.00**	

```
.
. *Other outcomes

.
. tab alive
```

Aliveatdisc harge	Freq.	Percent	Cum.
0	12	2.40	2.40
1	488	97.60	100.00
Total	500	100.00	

```
.
. swilk daysoncpap
```

Shapiro-Wilk W test for normal data

Variable	Obs	W	V	z	Prob>z
daysoncpap	500	0.65295	116.743	11.442	0.00000

```
.
. tabstat daysoncpap, stats (n mean med sd p25 p75 min max )
```

variable	N	mean	p50	sd	p25	p75	min	max
daysoncpap	500	2.1492	1.65	2.295002	.9	2.75	0	21.9

```
.
. swilk los
```

Shapiro-Wilk W test for normal data

Variable	Obs	W	V	z	Prob>z
losdays	500	0.64742	118.607	11.480	0.00000

```
.
. tabstat los, stats (n mean med sd p25 p75 min max)
```

variable	N	mean	p50	sd	p25	p75	min	max
losdays	500	8.398	6	7.981918	4	9	0	79

```
.
. tab ettever
```

ETTever	Freq.	Percent	Cum.
0	462	92.40	92.40
1	38	7.60	100.00
Total	500	100.00	

```
.
. swilk agem if alive==0
```

Shapiro-Wilk W test for normal data

Variable	Obs	W	V	z	Prob>z
agem	12	0.89682	1.724	1.061	0.14429

```
53 .
54 . tabstat agem if alive==0, stats (n mean med sd p25 p75 min max)
```

variable	N	mean	p50	sd	p25	p75	min	max
agem	**12**	**9.105**	**7.48**	**8.735429**	**.645**	**14.475**	**.26**	**27.11**

```
55 .
56 . tab wenticu
```

wenticu	Freq.	Percent	Cum.
0	**374**	**74.80**	**74.80**
1	**126**	**25.20**	**100.00**
Total	**500**	**100.00**	

```
57 .
58 . swilk los if wenticu==1
```

Shapiro-Wilk W test for normal data

Variable	Obs	W	V	z	Prob>z
losdays	**126**	**0.77056**	**23.009**	**7.044**	**0.00000**

```
59 .
60 . swilk los if wenticu==0
```

Shapiro-Wilk W test for normal data

Variable	Obs	W	V	z	Prob>z
losdays	**374**	**0.57974**	**108.946**	**11.127**	**0.00000**

```
61 .
62 . tabstat los if wenticu==1, stats(n mean med p25 p75 min max)
```

variable	N	mean	p50	p25	p75	min	max
losdays	**126**	**11.11111**	**8**	**6**	**13**	**0**	**55**

```
63 .
64 . tabstat los if wenticu==0, stats(n mean med p25 p75 min max)
```

variable	N	mean	p50	p25	p75	min	max
losdays	**374**	**7.483957**	**6**	**4**	**8**	**1**	**79**

```
65 .
66 . *Comparing those who went to PICU vs those who did not

67 .
68 . graph box agem, over(wenticu)

69 .
70 . graph box waz, over(wenticu)
```

```
.
. graph box daysoncpap, over(wenticu)

.
. graph box los, over(wenticu)

.
. tab wenticu alive, col row chi
```

Key
frequency<br>*row percentage*<br>*column percentage*

wenticu	Aliveatdischarge 0	1	Total
0	1<br>0.27<br>8.33	373<br>99.73<br>76.43	374<br>100.00<br>74.80
1	11<br>8.73<br>91.67	115<br>91.27<br>23.57	126<br>100.00<br>25.20
Total	12<br>2.40<br>100.00	488<br>97.60<br>100.00	500<br>100.00<br>100.00

Pearson chi2(1) = 28.8162   Pr = 0.000

```
.
. label define aliveatdischargelbl 0 "no" 1 "yes"

.
. label values aliveatdischarge aliveatdischargelbl

.
. label define wenticulbl 0 "no" 1 "yes"

.
. label values wenticu wenticulbl

.
. tab wenticu alive, col row chi
```

Key
frequency<br>*row percentage*<br>*column percentage*

wenticu	Aliveatdischarge no	yes	Total
no	1<br>0.27<br>8.33	373<br>99.73<br>76.43	374<br>100.00<br>74.80
yes	11<br>8.73<br>91.67	115<br>91.27<br>23.57	126<br>100.00<br>25.20
Total	12<br>2.40<br>100.00	488<br>97.60<br>100.00	500<br>100.00<br>100.00

```
          Pearson chi2(1) =  28.8162   Pr = 0.000

87 .
88 . tab wenticu alive,exact

           |   Aliveatdischarge
   wenticu |        no        yes |     Total
-----------+----------------------+----------
        no |         1        373 |       374 
       yes |        11        115 |       126 
-----------+----------------------+----------
     Total |        12        488 |       500 

           Fisher's exact =                 0.000
   1-sided Fisher's exact =                 0.000

89 .
90 . cs sex wenticu

                 |        wenticu         |
                 |   Exposed   Unexposed  |      Total
-----------------+------------------------+-------------
           Cases |        65         201  |        266
        Noncases |        61         173  |        234
-----------------+------------------------+-------------
           Total |       126         374  |        500
                 |                        |
            Risk |   .515873    .5374332  |       .532
                 |                        |
                 |      Point estimate    |    [95% Conf. Interval]
                 |------------------------+------------------------
 Risk difference |        -.0215601       |   -.1223951    .0792748 
      Risk ratio |         .9598831       |    .7909907    1.164837 
 Prev. frac. ex. |         .0401169       |   -.1648375    .2090093 
 Prev. frac. pop |         .0101095       |
                 +-------------------------------------------------
                               chi2(1) =     0.18  Pr>chi2 = 0.6749

91 .
92 . cs sex ettever

                 |        ETTever         |
                 |   Exposed   Unexposed  |      Total
-----------------+------------------------+-------------
           Cases |        16         250  |        266
        Noncases |        22         212  |        234
-----------------+------------------------+-------------
           Total |        38         462  |        500
                 |                        |
            Risk |  .4210526    .5411255  |       .532
                 |                        |
                 |      Point estimate    |    [95% Conf. Interval]
                 |------------------------+------------------------
 Risk difference |        -.1200729       |   -.2834967    .0433509 
      Risk ratio |         .7781053       |    .5309646    1.140279 
 Prev. frac. ex. |         .2218947       |   -.1402789    .4690354 
 Prev. frac. pop |          .016864       |
                 +-------------------------------------------------
                               chi2(1) =     2.03  Pr>chi2 = 0.1539
```

```
93 .
94 . cs chronic wenticu

                 |        wenticu         |
                 |   Exposed   Unexposed  |      Total
-----------------+------------------------+------------
           Cases |        32          59  |         91
        Noncases |        94         315  |        409
-----------------+------------------------+------------
           Total |       126         374  |        500
                 |                        |
            Risk |  .2539683     .157754  |       .182
                 |                        |
                 |      Point estimate    |    [95% Conf. Interval]
                 |------------------------+------------------------
 Risk difference |         .0962142       |    .0117087    .1807198
      Risk ratio |           1.6099       |    1.100955     2.35412
 Attr. frac. ex. |         .3788436       |    .0916973    .5752129
 Attr. frac. pop |         .1332197       |
                 +-------------------------------------------------
                               chi2(1) =     5.86  Pr>chi2 = 0.0155

95 .
96 . cs chronic ettever

                 |        ETTever         |
                 |   Exposed   Unexposed  |      Total
-----------------+------------------------+------------
           Cases |         9          82  |         91
        Noncases |        29         380  |        409
-----------------+------------------------+------------
           Total |        38         462  |        500
                 |                        |
            Risk |  .2368421    .1774892  |       .182
                 |                        |
                 |      Point estimate    |    [95% Conf. Interval]
                 |------------------------+------------------------
 Risk difference |         .0593529       |   -.0802388    .1989447
      Risk ratio |         1.334403       |    .7297424    2.440083
 Attr. frac. ex. |         .2506013       |   -.3703467    .5901778
 Attr. frac. pop |         .0247847       |
                 +-------------------------------------------------
                               chi2(1) =     0.83  Pr>chi2 = 0.3620

97 .
98 . cs aliveatdischarge wenticu

                 |        wenticu         |
                 |   Exposed   Unexposed  |      Total
-----------------+------------------------+------------
           Cases |       115         373  |        488
        Noncases |        11           1  |         12
-----------------+------------------------+------------
           Total |       126         374  |        500
                 |                        |
            Risk |  .9126984    .9973262  |       .976
                 |                        |
                 |      Point estimate    |    [95% Conf. Interval]
                 |------------------------+------------------------
 Risk difference |        -.0846278       |   -.1341925   -.0350631
      Risk ratio |         .9151453       |    .8668158    .9661695
 Prev. frac. ex. |         .0848547       |    .0338305    .1331842
 Prev. frac. pop |         .0213834       |
                 +-------------------------------------------------
                               chi2(1) =    28.82  Pr>chi2 = 0.0000
```

```
 99 .
100 . cs aliveatdischarge ettever
```

	ETTever Exposed	Unexposed	Total
Cases	**32**	**456**	**488**
Noncases	**6**	**6**	**12**
Total	**38**	**462**	**500**
Risk	**.8421053**	**.987013**	**.976**

	Point estimate	[95% Conf.	Interval]
Risk difference	**-.1449077**	**-.2613038**	**-.0285116**
Risk ratio	**.8531856**	**.7431551**	**.9795071**
Prev. frac. ex.	**.1468144**	**.0204929**	**.2568449**
Prev. frac. pop	**.0111579**		

chi2(1) = **31.48** Pr>chi2 = **0.0000**

```
101 .
102 . swilk daysoncpap if wenticu==1
```

Shapiro-Wilk W test for normal data

Variable	Obs	W	V	z	Prob>z
daysoncpap	**126**	**0.59833**	**40.282**	**8.302**	**0.00000**

```
103 .
104 . swilk daysoncpap if wenticu==0
```

Shapiro-Wilk W test for normal data

Variable	Obs	W	V	z	Prob>z
daysoncpap	**374**	**0.73142**	**69.627**	**10.065**	**0.00000**

```
105 .
106 . tabstat daysoncpap if wenticu==1, stats(n mean sd med p25 p75 min max)
```

variable	N	mean	sd	p50	p25	p75	min	max
daysoncpap	**126**	**2.283333**	**3.338233**	**1.3**	**.4**	**2.9**	**0**	**21.9**

```
107 .
108 . tabstat daysoncpap if wenticu==0, stats(n mean sd med p25 p75 min max)
```

variable	N	mean	sd	p50	p25	p75	min	max
daysoncpap	**374**	**2.104011**	**1.817582**	**1.7**	**1**	**2.7**	**0**	**16.7**

```
109 .
110 . kwallis los, by  (wenticu)
```

Kruskal-Wallis equality-of-populations rank test

**wenticu**	**Obs**	**Rank Sum**
**no**	**374**	**85252.00**
**yes**	**126**	**39998.00**

chi-squared =   **36.164** with **1** d.f.
probability =   **0.0001**

chi-squared with ties =   **36.490** with **1** d.f.
probability =   **0.0001**

111 .
112 . kwallis daysoncpap, by (wenticu)

Kruskal-Wallis equality-of-populations rank test

wenticu	Obs	Rank Sum
no	374	97363.00
yes	126	27887.00

chi-squared =   **6.868** with **1** d.f.
probability =   **0.0088**

chi-squared with ties =   **6.875** with **1** d.f.
probability =   **0.0087**

113 .
114 . kwallis los, by (ettever)

Kruskal-Wallis equality-of-populations rank test

ettever	Obs	Rank Sum
0	462	112901.00
1	38	12349.00

chi-squared =   **10.927** with **1** d.f.
probability =   **0.0009**

chi-squared with ties =   **11.025** with **1** d.f.
probability =   **0.0009**

115 .
116 . kwallis daysoncpap, by (ettever)

Kruskal-Wallis equality-of-populations rank test

ettever	Obs	Rank Sum
0	462	120966.00
1	38	4284.00

chi-squared =   **37.390** with **1** d.f.
probability =   **0.0001**

chi-squared with ties =   **37.425** with **1** d.f.
probability =   **0.0001**

117 .
